# Association between Suicidal Behaviors in Adolescence and Negative Emotions, the Level of Stress, Stress Coping Strategies and the Quality of Sleep

**DOI:** 10.3390/healthcare11030306

**Published:** 2023-01-19

**Authors:** Grażyna Cepuch, Agnieszka Kruszecka-Krówka, Patrycja Liber, Agnieszka Micek

**Affiliations:** Nursing and Midwifery Institute, Faculty of Health Sciences, Jagiellonian University Medical College, Kopernika Str. 25, 31-501 Krakow, Poland

**Keywords:** adolescents, anxiety, depression, aggression, stress, suicide

## Abstract

Suicidal behaviors in adolescents stem from complex processes deeply rooted in various spheres of life and functioning. The study was aimed at assessing the relationship between selected negative emotions, the quality of sleep, the level of perceived stress as well as stress coping strategies and suicidal ideation and attempts among high school students. The examined group consisted of adolescents aged 16–18 recruited by social-media groups in Poland. The study was based on a diagnostic survey with the application of CAWI method. The other research tools applied in the study included: Hospital Anxiety and Depression Scale—Modified, Perceived Stress Scale-10, How do you cope?—Questionnaire, Athens Insomnia Scale as well as the authors’ own questionnaire on suicidal ideation and suicide attempts. Anxiety, depression, aggression and insomnia as well as a high level of stress were statistically more common in girls than in boys (*p* < 0.001). The high level of negative emotions and insomnia, in turn, increased the risk of suicidal ideation (OR = 3.59, 95% CI: 2.13–6.06 and OR = 2.35, 95% CI: 1.60–3.46), as well as suicide attempts (OR = 6.29, 95% CI: 2.93–14.80 and OR = 3.29, 95% CI: 2.07–5.35). Additionally, high level of stress was associated with more than twice larger odds of suicidal ideation (OR = 2.26, 95% CI: 1.13–4.63). Significantly higher prevalence of suicidal ideation (*p* = 0.017) and suicide attempts (*p* = 0.016) was observed in girls. A high level of negative emotions and stress accompanied by a low quality of sleep are factors predisposing people to suicidal ideation and attempted suicide.

## 1. Introduction

Suicidal behavior as a social phenomenon in Poland is a problem which affects an increasing number of people of all ages. According to the data provided by the Polish Central Statistical Office (GUS) [1]—Local Data Bank, the number of suicide attempts among adolescents aged between 13 and 18 in 2018, 2019, 2020 and 2021 reached 746, 905, 814 and 1411, respectively. Out of all suicide attempts in the analyzed years, 417 ended in death in the 13–18 age group.

The research into suicidology conducted among young people can only make us aware of the actual scale of the problem, which requires further analysis and exploration. Suicidal ideation and attempts in adolescents stem from complex processes deeply rooted in various spheres of life and functioning. Numerous causes of suicide include anxiety disorders, which are diagnosed in a significant percentage of adolescents making a suicide attempt [2,3,4,5], as well as depressive disorders [5,6,7,8]. Note that early life trauma, neglect, inadequate parenting, and ongoing stress are among the environmental factors that contribute to the higher likelihood of developing anxiety, depression, and other stress-related disorders [5]. The predictions about teenagers with depressive disorders seem to be particularly worrying. The observations which have been made so far lead to an assumption that a significant percentage of these adolescents are likely to attempt suicide in adulthood [9,10]. Adolescents are particularly prone to depressive and anxiety disorders due to dynamic biological and emotional changes (e.g., emotional lability and different stimuli perception) as well as social ones (e.g., perceived lack of support, impulsiveness of actions), which make it difficult for young people to adapt to new demands of the world around them [11].

The relationship between suicidal attempts and aggressive disorders is not a matter of controversy either. A moderate correlation between aggression and suicidality among adolescents is indicated; however, the average effect size might be smaller or larger depending on methodological features of individual research reports [12,13,14]. Hartlej et al. [15] point out that not only the level of aggression but also its type (reactive or proactive aggression) is of key importance in order to determine suicidal behaviors. Even though behavioral manifestations of reactive and proactive aggression often look the same, their functions, correlates and results are different. Koyama et al. [16] also point out that genetic susceptibility to impulsive aggression and difficult experiences remain a significant predictor for suicide ideation over and above the youth’s level of anxiety and depression.

Furthermore, an important predictor of suicide is also sleep disorders, which are more and more common in industrialized societies and are also diagnosed in children and adolescents [17,18,19]. Moreover, scientific reports indicate a two-way relationship between depression and anxiety and sleep disorders [20,21]. Sleep disturbances could be indicative of future mental health problems, particularly depression, in this young sample, which highlights the potential of harnessing sleep as a tool to mitigate the persistence of depression across early-adolescence and potentially prevent an adolescent onset of major depressive disorders [22,23,24]. Sleep disorders, especially in combination with other disorders, may have serious consequences: not only social ones but, most of all, consequences for the adolescents suffering from the disease [25,26,27].

Due to the nature of adolescence, the implications associated with this period can generate high levels of stress [11,28,29]. It becomes more and more important to choose the right strategy of coping with stress, which is understood as an ability to tolerate stress, control it or reduce its level [30,31]. Experienced stressors and polyvictimization are significant predictors of the risk of developing somatoform and emotional disorders and, consequently, suicidal ideation and attempts [32,33,34]. Adolescents’ inability to apply adaptive strategies has a negative impact on the quality of their mental health [33,35,36].

Taking into account the aforementioned premises, a study was conducted in the population of teenagers, the aim of which was to assess the quality of sleep, level of depression, anxiety, aggression, level of stress and stress coping strategies as potential factors associated with suicidal ideation and attempts in the population of adolescents.

## 2. Materials and Methods

### 2.1. Study Group

#### 2.1.1. Study Design

The study was designed as a cohort study in a population of teenagers aged between 16 and 18, in order to assess the prevalence and intensity of depression, anxiety and aggression, level of stress and stress coping strategies as well as the quality of sleep within the previous three months, as potential risk factors of suicidal ideation and attempts. The survey was designed for respondents of European origin, non-emigrants who declared to have a good socio-economic status of their family and resided in rural, small-town and urban environments.

In order to achieve the assumed goal of the study, the following research questions were formulated:

1. What is the level of anxiety, depression, aggression, stress, sleep quality and coping strategies in the studied group of adolescents according to sex?
2. Does sex significantly differentiate the prevalence of suicidal ideation and attempts?
3. Which factors, independently of the others, are related to suicidal ideation/attempts and to what extent?

The results of the study were described following the STROBE guidelines for cohort studies [37]. The study was fully anonymous and voluntary, taking into account personal data security and protection. It was not possible to complete the questionnaire twice. The questionnaire included information for a patient over 16 to 18 years of age, attending high school, an informed consent form and a personal data processing form, and there was no opportunity to proceed to the next stage without confirming that this content has been read and understood. The study was planned and conducted following the relevant legal recommendations and ethical principles of the Declaration of Helsinki. The protocol of the study was approved by the Bioethics Committee of the Jagiellonian University in Krakow (No. 1072.6120.4.2021 issued on 20 January 2021).

#### 2.1.2. Sample, Setting & Data Collection

The study group included 793 students aged between 16 and 18. Respondents were randomly recruited from the most popular social-media groups in Poland, which seemed to be the safest form of data collection in the time of the pandemic (from March 2021 to May 2021). The selected period of time was related to restrictions resulting from SARS-CoV-2 pandemic. Respondents had no opportunity to participate in stationary classes and their access to extracurricular activities was limited. 

Participants involved in a research process belonged to a cohort of male and female teenage population living both in the city and in the country and attending high schools. 

There were the following inclusion criteria for the study declared in the questionnaire: attending high school, no learning difficulties at school, good social functioning, including no problems with peers and being raised in well-functioning families. Additionally, respondents’ subjective sense of well-being was taken into account. 

The exclusion criteria included respondents’ declaration of a mental disease, the incidence of chronic sleep disorders requiring treatment in the course of the study, psychological support provided to a respondent, chronic somatic diseases as well as disability (temporary/permanent) or lack of consent for participation in the study. 

The study was based on a diagnostic survey with the application of CAWI (Computer Assisted Web Interview) method. CAWI is part of methodology based on a survey delivered to the respondent via a link in a panel or on a website. The CAWI method is considered one of the most economical ways to collect survey data, as it does not require interviewers, equipment, or additional tools. The link to the forms was created on the Microsoft survey platform, purchased and provided by Jagiellonian University. The link to the form was sent to groups of only high school students, along with information about the purpose of the study and a request to fill in the form. The process of recruitment was carried out by posting adverts on social media sites along with sharing requests, thus allowing the transmission of the questionnaire to someone else. Exemplary groups in which the information about the possibility of participating in the study was posted were thematic groups related to science, entertainment or interests. The questionnaire included information for adolescents, an informed consent form and a personal data processing form and there was no possibility of proceeding to the next stage without confirming that this content had been read and understood. The questionnaires did not include questions which might imply an impact of the pandemic on the functioning of individuals. It was not possible to complete the questionnaire twice.

Statistical analysis was performed taking into account only the questionnaires in which all tool sheets were fully completed.

Knowing that suicidal ideation and attempts in adolescent can vary between sexes, we calculated, a priori, a sample size needed to detect the difference between the percentage of men and women who experienced suicidal attempts.

We calculated, a priori, a minimal sample size needed to achieve adequate statistical power (80%) and detect the relation between covariate and suicidal attempts during the whole life with an effect given by the size of the parameter OR equal to 1.6. Under the assumption of anticipated probability that at least one suicide attempt during the whole life equals 20% among teenagers aged 16–18 and Type I error is set at the level 0.05, we conclude that 788 persons would be sufficient for a wide class of distributions of tested covariates (binomial, normal, etc.).

#### 2.1.3. Participants and public involvement

The study was conducted in a group of adolescents who were the only source of information.. The information from the author’s questionnaire on suicidal ideation and attempts was only the information declared by the adolescents participating in the study.

### 2.2. Description of Research Tools

#### The Study Employed

− Hospital Anxiety and Depression Scale (HADS), original version developed by: Zigmond and Snaith [38], Polish version adapted by: Majkowicz, et al. [39]. The HADS-M scale is used for screening and contains three independent subscales: depression, anxiety and aggression, consisting of a total of 16 questions (each can be scored from 0 to 3 points: depression subscale (7 questions)—the maximum number of points is 21, anxiety subscale (7 questions)—the maximum number of points is 21 and aggression subscale (2 questions)—the maximum number of points is 6.

The interpretation of the number of points for each subscale is as follows: no disorders: 0–7 pts.—depression/anxiety subscale, 0–2 pts.—aggression subscale; borderline states: 8–10 pts.—depression/anxiety subscale, 3 pts.—aggression subscale; observed disorders: 11–21 pts.—depression/anxiety subscale, 4–6 pts.—aggression subscale.

In total, a subject can score a maximum of 48 points (this is the sum of the maximum number of points from each subscale); relating the percentage scores for each subscale, we obtained the following results (no disorders: 0–33.33%, borderline states: 33.34–47.62%, observed disorders: 47.63–100%). As the sum score, individual conditions can be defined as: 0–16 pts.—no disorders, 17–22 pts.—borderline states, 23–48 pts.—presence of disorders. Validation studies of the basic and modified versions of the HADS scale demonstrated its satisfactory reliability and accuracy [39,40]. The Spearman’s rank correlation coefficient calculated for the test items and the overall score of a given subscale was statistically significant at least at the *p* = 0.01 level and ranged from 0.41 to 0.76. The HADS is a widely used method of measuring anxiety and depression both in psychiatric practice and in the study of mentally healthy individuals in whom, for some reason, emotional state must be assessed [39,40]. For the purposes of the present study, each subscale was analyzed separately rather than in total.

− The Perceived Stress Scale—10 (PSS-10), developed by Cohen, et al. [41] and adapted to the Polish version by Juczyński and Ogińska-Bulik [42]. The scale can be used for screening to identify individuals qualifying for psychological or medical help. The PSS- 10 stress severity index is considered a good predictor of physical and mental health. The scale consists of 10 questions referring to respondents’ subjective feelings connected with problems and personal experience, behaviors and coping strategies which are assessed on a 5-point scale ranging from 0 (never) to 4 (very often). Before calculating the general indicator of the intensity of perceived stress, changes are introduced in the scores for positively formulated questions (4, 5, 7, 8) following the rule: 0 = 4; 1 = 3; 3 = 1; 4 = 0. The total score is the sum of all scores and ranges between 0 and 40; the higher the score, the higher the intensity of perceived stress. The general index is transformed into standardized units and interpreted according to the proprieties characterizing a sten scale. The score ranging from 1 to 4 stens is considered to be low, from 5 to 6 stens—average and from 7 to 10 stens—high. The score of 10 on the PSS scale is an indicator of assessing one’s own life situation as stressful, that is unpredictable, beyond control and excessively burdensome.
− How do you cope? (Jak sobie radzisz?) Questionnaire (JSR)—developed by Juczyński and Ogińska-Bulik [42] following the paradigm of the research by Lazarus and Folkman [43], according to which human actions usually have two functions—they aim to solve a task or regulate emotions. The authors distinguished two forms of coping in a stressful situation. The first is focused on solving the problem, the second one on emotions.

Juczynski’s scale captures both the dispositional characteristic of each individual repertoire of strategies, and situational ways of coping with a stressful situation, i.e., the strategies used in a specific, previously experienced situation [42]. The “How do you cope?” scale consists of two parts: the first—designed to measure dispositional ways of coping with stress (DCS), and the second—to measure situational ways of coping with stress (SCS) in a difficult situation. This scale examines whether the respondent tends to cope in an active way (ACS—active coping with stress), focuses on emotions (EF—emotional focus) or seeks social support (SSS—seeking social support). Active coping with stress aims to take control over stress and, consequently, change the situation, using the individual’s own skills and resources (the emphasis is on the task or planning to solve the problem). A coping strategy focused on oneself and one’s own emotions is directed at reducing unpleasant emotional tension. The style focused on seeking social support is concentrated on the emotions of the individual, and seems particularly important in relation to the behavior of children and adolescents in stressful situations [42]. Each of the two parts of the scale consists of nine questions. The answers are given on a 5-point scale, on which in the first part of the questionnaire ‘1′ means ‘hardly ever’ and ‘5′ means ‘almost always’ and in the second part of the questionnaire ‘1′ means ‘definitely no’ and ‘5’ means ‘definitely yes’. The values obtained on this scale are converted into sten norms. Following the Polish standardization studies a satisfactory level of reliability and accuracy was confirmed. The reliability of the scale tested for children and adolescents—Cronbach’s alpha coefficient was 0.86 [42].

− Athens Insomnia Scale (AIS)—Polish version was used to assess insomnia [44]. The tool contains eight questions about various insomnia symptoms (range 0–24, higher scores indicating worse sleep). The first five positions allow for the identification of such symptoms of sleep disorders as difficulty in falling asleep, waking up at night, waking up early in the morning, duration, and the quality of sleep. The other three questions concern daily functioning and assess well-being, physical and psychological efficiency, as well as the feeling of drowsiness. The symptoms are scored on a scale of 0–3 points (where 0 means the absence of any symptoms and 3—severe intensity of symptoms) only if they occurred at least three times a week during the last month. Obtaining a minimum of 8/24 points for a patient indicates the likelihood of insomnia. The AIS can be used, both in clinical practice and research, as a tool for measuring the severity of sleep problems and as a screening tool for diagnosing insomnia in adolescents and adults [44,45].
− Questionnaire designed by the authors themselves and consisting of two items connected with the incidence of suicidal ideation and attempts. The survey included definitions of the terms: suicidal ideation and suicide attempts. Suicide attempts were defined as a situation when one tries to end one’s life but does not succeed. Suicidal ideation were defined as fantasies and thoughts about suicide, as well as desires to commit suicide. The questions are answered on a 2-point scale, where ‘0′ means ‘never’ and ‘1′ means ‘at least once’. The scale was not standardized and, therefore, before the survey its participants were instructed to answer the questions in a completely subjective manner deciding on their own what dimension a particular item’s distractor would have for them.

### 2.3. Statistical Analysis

Due to different suicidal attitudes among male and female adolescents, descriptions of the sample and univariate comparisons were presented separately by sex. The characteristics of the examined group was depicted as counts and percentages in the case of qualitative variables, whereas the quantitative measurements given by scores of points or stens were presented by medians and quartiles. The chi-square test was employed to examine the differences between genders and suicidal thoughts and behaviors in the distribution of categorized variables such as the level of negative emotions, stress and quality of sleep. In turn, the U Mann–Whitney test and Kruskal–Wallis ANOVA rank test were used to analyze the significance of differences between the aforementioned groups in the assessment of quantitative features (e.g., particular stress coping strategies applied by adolescents). Relationships between coping strategies were measured by means of Spearman’s rank correlation coefficient. The Guilford’s classification was employed to assess the strength of correlations. Moreover, the multivariable logistic regression was applied to examine the association between potential risk factors and suicidal ideation and attempts. The study presented two models best fitted to the data and chosen based on the preliminary bilateral analyses. They included the most important risk factors of suicidal attempts and ideation as well as potential confounders. Taking into account possible differences between males and females, the first-order interactions of the main explanatory variables with gender were verified, and, after confirming lack of significance, were not incorporated in the final analyses so as not to reduce the power. As a kind of sensitivity analyses, two approaches were showed, one considering HADS-M, PSS and AIS as categorical variables and the second including them in logistic regression as continuous variables. Two-tailed significance level was set at *p* < 0.05. The analysis was conducted applying TIBCO Statistica program and R Software for Windows (R Foundation for Statistical Computing, Vienna, Austria, version 4.0.4).

## 3. Results

### 3.1. Characteristics of the Study Group

The study group consisted of 793 high school students aged between 16 and 18. The girls made up 78.4% (n = 622) of respondents. Furthermore, 40.6% of students (n = 322) lived in rural environment—Table 1. 

The assessment of depression, anxiety and aggression according to HADS scale showed higher levels in females as compared to males (26% vs. 14.6% for depression, 53.9% vs. 29.2% for anxiety, 61.7% vs. 46.2% for aggression), which was indicative of more frequent incidence of disorders in young women. In the examined group of adolescents, high scores were observed mainly on the aggression subscale (58.4%, n = 463), less frequently on the anxiety subscale (48.5%, n = 385) and the least frequently on the depression subscale (23.6%, n = 187).

The total high score obtained from all the subscales indicated that the incidence of disorders was almost twice as high in girls as in boys (49.2%, n = 306) vs. 26.3%, n = 45). Most high school female students and almost a half of high school male students obtained a high score on the scale of perceived stress (74.4%, n = 463 vs. 48.5%, n = 83, *p* < 0.001).

A statistically significant difference between males and females was observed in stress coping strategies, in terms of disposition in EF and SSS strategies and situationally in ACS and EF strategies. In the group of girls, the highest scores were recorded successively in EF, ACS and SSS strategies, whereas in the group of boys they were recorded in ACS, EF and SSS strategies. The same trends were observed both in the dispositional and situational part of the test (Table 1). 

In the examined group of teenagers, high scores on AIS scale, which could indicate the incidence of insomnia, were significantly more frequent in girls (54.0% vs. 34.5%, *p* < 0.001). The incidence of suicidal thoughts was declared by 64.8% of girls (n = 403) and 54.4% of boys (n = 93). As many as 24.1% (n = 150) of female and 15.2% (n = 26) of male high school students had at least one attempted suicide episode. The incidence of suicidal thoughts and suicide attempts was significantly more common in girls than in boys (*p* = 0.017 and *p* = 0.016, respectively (Table 1).

### 3.2. Examined Emotions, Sleep Quality and Stress Coping Strategies vs. Suicidal Ideation and Attempts

**Figure 1:**
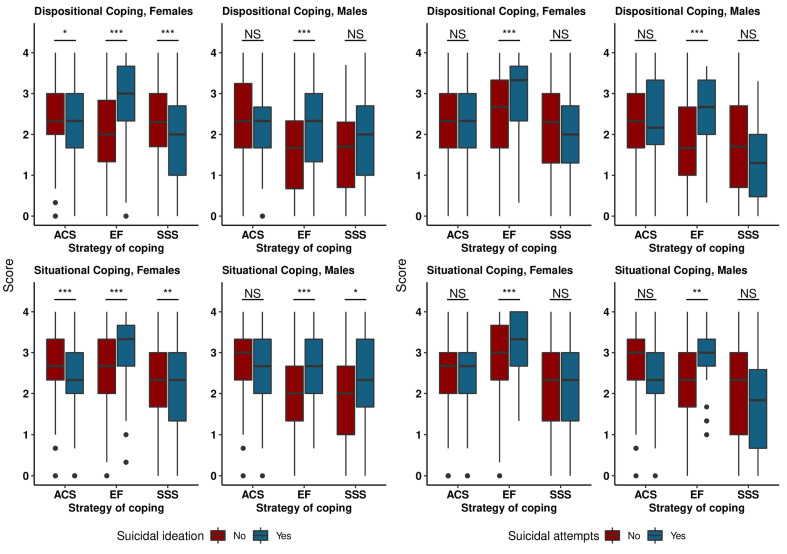
Comparison of level/intensity of coping strategies across suicidal ideation categories and suicidal attempts categories and by sex. Note: *** *p* < 0.001, ** *p* < 0.01, * *p* < 0.05.

A strong significant relationship was observed between suicide attitudes and the intensity/level of depression, anxiety and aggression, stress, quality of sleep and some specific coping strategies. In both gender groups, each of the scores of HADS-A anxiety, HADS-R aggression, HADS-D depression, PSS and AIS was consistently higher in adolescents who declared suicidal ideation as well as suicidal attempts (Table 2). No association was found between the intensity of ACS and SSS strategies and the incidence of suicidal attempts either in the female or male group. Lower ACS and SSS scores were obtained in the group of girls who admitted to having suicidal thoughts, whereas in the case of boys the results were inconsistent and usually insignificant (Figure 1, Table 2). However, statistically significantly higher scores were observed on dispositional and situational scales of EF strategy in people of both genders who experienced suicidal ideation or suicide attempts.

To select the best risk factors of suicide attitudes for inclusion in a multivariable logistic regression model and avoid multicollinearity by incorporating redundant variables, we additionally checked the correlations between the intensity of specific strategies of coping. In both genders there was a high positive correlation between the scores of a given dispositional coping strategy and a corresponding situational coping strategy (Spearman rank correlation coefficients of 0.46 and 0.59 for ACS, 0.69 and 0.61 for EF and 0.74 and 0.70 for SSS for males and females, respectively). Additionally, there was a significant positive correlation of ACS with SSS in both genders and both dispositional and situational strategies (Table 3).

### 3.3. Factors of Suicidal Ideation and Suicide Attempts—Multivariable Logistic Regression Analysis

Taking into account an analysis of correlations between all coping strategies as well as the results of univariate comparisons between coping strategies and both suicidal attempts and thoughts (ACS and SSS in both dispositional coping strategy and situational coping strategy were not associated with suicidal attempts in univariate comparisons in either gender nor with suicidal thoughts in males), in multivariable logistic regression analysis firstly only EF and SSS were included in fully adjusted regression models. However, because SSS in all analyses was insignificant in final models only, EF dispositional strategy was incorporated to reduce multicollinearity and increase power and ensure the most parsimonious model. Regardless of other factors such as a place of living, negative emotions and stress, AIS and EF dispositional strategies were associated with suicidal ideation and only HADS, AIS and EF dispositional strategies were associated with suicide attempts. Persons with symptoms of HADS had almost 4-fold larger odds of suicidal ideation and more than 6-fold larger odds of suicide attempts compared with a group without any signs of negative emotions (OR = 3.59, 95% CI: 2.13–6.06 and OR = 6.29, 95% CI: 2.93–14.80, respectively). When three dimensions of HADS were simultaneously incorporated in the model as continuous variables, only anxiety and depression increased the odds of both outcomes and the increment of size effect was from 6% to 13% with one unit in the fully adjusted model (model 1, Table 4). Persons with sleeping disorders had more than twice larger odds of suicidal thoughts (OR = 2.35, 95% CI: 1.60–3.46 in fully adjusted model) and more than threefold larger odds of suicidal attempts (OR = 3.29, 95% CI: 2.07–5.35 in fully adjusted model). In a model with continuous score of AIS, the chance of suicidal thoughts and attempts increased about 8% and 14%, respectively, with increments of AIS per 1 point (OR = 1.08, 95% CI: 1.03–1.14 and OR = 1.14, 95% CI: 1.08–1.20, respectively). Only categorised PSS showed a significant relationship with the frequency of occurrence of both outcomes. The odds of suicidal thoughts were almost twice and more than 3.5 times larger in medium and high level of PSS group compared with low (OR = 1.82, 95% CI: 1.11–2.99 and OR = 3.59, 95% CI: 2.13–6.06, respectively). Even stronger effect, although with wider confidence intervals, was observed for suicide attempts (OR = 2.33, 95% CI: 1.03–5.60 OR = 6.29, 95% CI: 2.93–14.80, respectively). Linear effect of PSS was on the boundary of significance but showed the same trend.

Finally, EF dispositional increment per 1 was associated with 25%–35% larger odds of suicidal thoughts, depending on the considered model, whereas the effect on suicide attempts, however insignificant in fully adjusted models, in the model adjusted to HADS, PSS, AIS and place of living, revealed the same direction with 23% higher odds of occurrence of the outcome (Table 4).

## 4. Discussion

Suicidal ideation and attempts are phenomena which result from experiencing various adverse stress-inducing situations. It was the number of adolescents who declared suicidal thoughts and suicide attempts that was the matter of particular concern in the authors’ study, especially because the examined group reported no disfunctions in their family life, no learning difficulties and perceived themselves as well-functioning in both physical and social aspect. Cha et al. [46] observe that adolescents report a higher proportion of suicidal ideation and attempts than adults do, which makes this developmental period a critical one and, therefore, requiring early identification of factors which contribute to suicidal behaviors.

Suicidal ideation is considered to be one of the first stages which may lead to committing suicide [47]. It should be noted that suicidal ideation, often referred to as suicidal thoughts, is a broad term. In fact, there is no universally accepted consistent definition, which presents a continuous challenge for clinicians and researchers. A very interesting concept of the risk of developing suicidal ideation and attempts was presented by Ho et al. [48], who emphasized that suicidal thoughts and behaviors in adolescents result from their pathological reactions to social stressors. However, it should be noted that stress regulation systems are still maturing and the incidence of an unpredictable difficult situation can lead to suicidal actions in individuals who were potentially not considered at risk of suicide. The authors cited above argued that alterations in brain circuits, comprised of connections among the amygdala, hippocampus, striatum, anterior cingulate cortex, medial prefrontal cortex and orbitofrontal cortex that underlie social cognition, emotion regulation and impulse control are shaped by puberty-related changes in sex hormones. 

In the conducted study, suicidal ideation was declared by 1/3 of female respondents and 1/5 of male respondents, whereas over 1/5 of all surveyed high school students admitted to having at least one suicidal attempt. These results seem to be in line with the results obtained by other authors [49,50,51].

In the group of adolescents, anxiety, depression and aggression are considered to be predictors of suicide attempts [3,5,52]. Despite numerous undertaken preventive measures, the prevalence of depressive [8,53,54,55] and anxiety disorders [5,56] among young people is still widespread, which was also proved by the results of this study. This phenomenon is common particularly in females. Additionally, aggression was significantly more prevalent among females, which, however, is not confirmed by the reports of other authors, who indicated more frequent incidence of aggressive disorders among boys [57,58]. An attempt to explain why male gender is more often identified with manifestation of aggression was made by Eliot [59], who at the same time emphasized that no human being is born inherently programmed for violence but learns to balance prosocial and antisocial impulses following specific resources and requirements of the environment. 

Another variable related to adolescents’ functioning and crucial in indicating suicidal behaviors is stress and its level [60]. In the group of teenagers examined in our study, the level of stress was relatively high and, as in the case of other assessed negative emotions, its higher level was correlated with female gender. Scientific research confirms both the prevalence of stress in adolescents and its varied severity [61,62,63,64,65]. The differences in the level of stress can be explained not only by the characteristics of the examined groups but also by cultural difference which may determine how negative emotions are experienced and dealt with. 

The assessment of the quality of sleep in the examined group produced disturbing results. Although the group subjectively rated the quality of their sleep as good, the study found that more than a half of the respondents, mostly girls, suffered from sleep disorders. These results are similar to those obtained by other authors, which confirms that a significant percentage of the adolescent population suffer from insomnia [18,66,67,68,69,70,71]. It was observed that sleep disorders are not strong unidimensional predictors of suicidal behaviors [72]; however, when accompanied by other factors, their importance increases [69,73,74]. Assessing adolescents for sleep disorders should be prioritized, given their strong association with depression [75]. 

The results of the current study also confirm the relationship between respondents’ gender and their choice of a coping strategy. The most frequently selected stress coping strategies in the female group were focusing of emotions, active coping, and then seeking social support, which is consistent with other reports [31,65,76]. In the male group, in turn, an active model of reaction was the dominating one. The active coping strategy, which was frequently chosen in this group, emphasizes planned problem solving. Focusing on the problem means better control over stress, and, consequently, results in changing a particular situation. The ability to perceive a difficult situation as a challenge and to consciously experience positive emotions may protect against depressive mood disorders and allow for efficient stress management [77]. Non-adaptive strategies of coping with stress, which were dominating ones in the case of female group, might imply a greater vulnerability to mental disorders, and consequently, worse quality of life as well as a higher tendency to engage in suicidal behaviors. 

In the current study, the relationship between the intensity of anxiety and depression and suicidal attempts in the group of adolescents was also confirmed. Additionally, a significant relationship was observed between declaring suicidal thoughts and anxiety and depression. Avenevoli [53] and Miller [55] noticed that suicide rates have increased for both genders but in the case of girls the increase was more significant, which was not proven in our study. It is a matter of concern that although the prevalence of depression has increased across all age groups, the increase among adolescents has outpaced that among adults [55]. 

The results of the current study showed a positive correlation between sleep disorders and the prevalence of suicidal ideation and suicide attempts, similarly to the results obtained by other authors [68,78,79,80,81,82].

Suicidal attempts in adolescents were not connected with their level of stress. However, a relationship was shown between suicidal ideation and suicide attempts and adolescents’ choice of coping strategies focused on emotions, which is confirmed by the results obtained by other authors [83,84]. In the study by Miller et al. [55], Mars et al. [85] and Beatie et al. [86], a high level of stress and sleep disorders [85,86], as well as a high level of negative emotions [55] were significantly associated with the incidence of suicidal thoughts. Additionally, female gender is considered to be a key predictor of suicidal attempts in adolescents [87]. In the current study, female gender was associated with negative emotions, higher level of stress, less efficient coping strategies and poor sleep quality. These factors determined the occurrence of suicidal ideation and attempts potentially indicating that they may act as mediators of the relationship between gender and suicidal behavior. 

## 5. Strengths, Limitations and Implications of Research

The study had several limitations which might have affected its results; therefore, the need for caution must be stressed when comparing it to other reports. The timing of the study coinciding with SARS-CoV-2 pandemic might have determined adolescents’ functioning in a direct and indirect way, including their mental health. The changes generated by the epidemiological situation such as online learning, ignoring daily sleep patterns of waking up and falling asleep, lack of opportunities for contact with their peers, limited access to natural light and the possibility of more frequent use of electronic devices emitting blue light during evening hours, may have exacerbated the phenomenon of sleep disorders in the examined group of adolescents. Another limitation was the selection of self-report scales, which were only a screening tool and did not check actual health status. It should be particularly taken into consideration when comparing the scores related to aggression. The HADS-M, despite its high accuracy, includes only two items concerning aggression, which may make it less reliable in comparison to other scales. Moreover, the HADS-M questionnaire used in the current study is based only on the respondents’ declarations, so it cannot be used to diagnose disorders. However, due to its high sensitivity, it can be used for pilot identification of symptoms indicating a growing trend of depressive disorders, anxiety and aggression among adolescents. Thus, only a general trend of the examined phenomena and disfunctions can be identified among adolescents with some probability, without taking into account their actual medical condition. The study was conducted in a group including only high school students who came from well-functioning families and declared no school-related problems. It cannot be referred to the general population as other types of secondary schools were not included. The survey was conducted with the application of the CAWI method and, as the interviewers were not present during the survey, it was not possible to control the external factors affecting its course, which could distort respondents’ answers. Additionally, the difference in the number of female and male respondents in the sample group may have implied differences in the results obtained.

In contrast, the study’s strengths include a comprehensive assessment of suicide risk factors among a relatively large group of adolescents in such significant dimensions as depression, anxiety, aggression, quality of sleep and stress coping strategies. In addition, the study was conducted in a cohort of adolescents without psychiatric or psycho-emotional disorders or family disfunction, which limited the probability of distorting the results or their interpretation. Moreover, the regression model included multiple variables which could function as potential confounding factors and the most important risk factors were examined both as quantitative characteristics, which could verify their “linear” effect, and as categorized variables.

Taking into account aforementioned disturbing trends within the adolescent population, even without school or family burdens, it seems particularly important to undertake further research to expand our knowledge regarding the impact of the variables studied. Further research in this area should aim at the selection of equal study groups as far as gender is concerned, the inclusion of respondents from other types of secondary schools and with different family backgrounds, the application of a more meaningful method of measurement, primarily in the context of aggression, and the inclusion of an assessment of the impact of the pandemic on particular correlates of suicidal behavior. Adolescents who are considered potentially healthy and well-functioning are also at risk of experiencing suicidal ideation and attempts, due to developmental characteristics and the consequences associated with this period of life.

The results of the study should be available for people who have direct contact with adolescents, such as nursing and medical teams working in medical and educational institutions, teachers and parents, to help them identify adolescents who are most at risk of attempting suicide in the future and to develop prevention and intervention strategies necessary for adolescents during a suicide crisis. Although in Poland there is a Working Group for Prevention of Suicide and Depression at the Department of Public Health of the Ministry of Health, no national suicide prevention program has been developed so far; the need for which has already been reported.

## 6. Conclusions

A significant percentage of adolescents have suicidal thoughts and make suicide attempts. Adolescents exhibit high and moderate levels of depression, anxiety and aggression as well as high level of stress correlated with poor quality of sleep especially in females. Females most often choose coping strategies focused on emotions, whereas males tend to choose strategies based on active coping. The level of sleep quality is highly correlated with the intensity of stress levels and moderately correlated with the choice of coping strategies based on emotions and the prevalence of suicidal tendencies in young people. Adolescent suicide attempts are linked with depression, anxiety, aggression and a low quality of sleep. Additionally, gender may have an indirect influence on the aforementioned factors, moderating the examined relationships with the incidence of suicidal thoughts, although to a lesser extent than in the case of suicide attempts.

## 7. Search Strategy and Selection Criteria

The choice of research strategy was aimed at assessing the impact of negative emotions, stress, coping strategies and the quality of sleep on the incidence of suicidal behaviors in high school students. The report was prepared following STROBE guidelines and English-language articles published between 2016 and 2021 and available in Google Scholar and PubMed databases. The search was completed in December 2021. The keywords applied in the search included suicide, adolescents, depression, aggression, anxiety, coping strategies, suicide attempts, suicidal thoughts, sleep disorders, sleep quality, suicidal tendencies, children, adolescents, high school students, mental disorders.

## Figures and Tables

**Table 1:** Characteristic of study sample across sex categories (N = 793).

Variable & Categories		Sex		*p* *
	Female	Male	Total	
	(N = 622)	(N = 171)	(N = 793)	
Socio-demographic factors				
Age, n (%)				
16 years old	220 (35.4)	55 (32.2)	275 (34.7)	0.721
17 years old	195 (31.4)	55 (32.2)	250 (31.5)	
18 years old	207 (33.3)	61 (35.7)	268 (33.8)	
Place of residence, n (%)				
City	363(58.4)	108 (63.2)	471 (59.4)	0.297
Country	259 (41.6)	63 (36.8)	322 (40.6)	
Having siblings, n (%)				
No	82 (13.2)	14 (8.2)	96 (12.1)	0.203
Yes	540 (86.8)	157 (91.8)	697 (84.2)	
Emotional factors				
HADS				
Anxiety, n (%)				
No symptoms	156 (25.1)	80 (46.8)	236 (29.8)	<0.001
Boundary state	131 (21.1)	41 (24.0)	172 (21.7)	
Disorder	335 (53.9)	50 (29.2)	385 (48.5)	
Aggression, n (%)				
No symptoms	161 (25.9)	71 (41.5)	232 (29.3)	<0.001
Boundary state	77 (12.4)	21 (12.3)	98 (12.4)	
Disorder	384 (61.7)	79 (46.2)	463 (58.4)	
Depression, n (%)				
No symptoms	334 (53.7)	123 (71.9)	457 (57.6)	<0.001
Boundary state	126 (20.3)	23 (13.5)	149 (18.8)	
Disorder	162 (26.0)	25 (14.6)	187 (23.6)	
PSS				
Stress level, n (%)				
PSS—SUM < 14	47 (7.6)	38 (22.2)	85 (10.7)	<0.001
PSS—SUM ≥14 and SUM ≤19	112 (18.0)	50 (29.2)	162 (20.4)	
PSS_SUM>19	463 (74.4)	83 (48.5)	546 (68.9)	
AIS, n (%)				
AIS-SUM ≤ 8	286 (46.0)	112 (65.5)	398 (50.2)	<0.001
AIS-SUM > 8	336 (54.0)	59 (34.5)	395 (49.8)	
Suicide attempts, n (%)				
No	472 (75.9)	145 (84.8)	617 (77.8)	0.017
Yes	150 (24.1)	26 (15.2)	176 (22.2)	
Suicidal ideation, n (%)				
No	219 (35.2)	78 (45.6)	297 (37.5)	0.016
Yes	403 (64.8)	93 (54.4)	496 (62.5)	
PSS—SUM, q2 (q1–q3)	25.00 (19.00–29.00)	19.00 (14.00–24.50)	24.00 (18.00–28.00)	<0.001
PSS—STEN, q2 (q1–q3)	8.00 (6.00–9.00)	6.00 (5.00–8.00)	8.00 (6.00–9.00)	<0.001
AIS—SUM, q2 (q1–q3)	9.00 (6.00–13.00)	6.00 (4.00–10.00)	8.00 (5.00–13.00)	<0.001
JSR				
Dispositional stress coping strategies,				
q2 (q1–q3)				
ACS	2.33 (1.67–3.00)	2.33 (1.67–3.00)	2.33 (1.67–3.00)	0.902
EF	2.67 (2.00–3.33)	2.00 (1.00–2.67)	2.67 (1.67–3.33)	<0.001
SSS	2.00 (1.30–2.70)	1.70 (0.70–2.30)	2.00 (1.00–2.70)	<0.001
Situational stress coping strategies,				
q2 (q1–q3)				
ACS	2.67 (2.00–3.00)	2.67 (2.00–3.33)	2.67 (2.00–3.33)	0.021
EF	3.00 (2.33–3.67)	2.33 (1.67–3.00)	3.00 (2.33–3.67)	<0.001
SSS	2.33 (1.33–3.00)	2.00 (1.00–3.00)	2.33 (1.33–3.00)	0.306

* *p*—*p*-value based on Chi-square test of independence or Mann Whitney U test.

**Table 2:** Comparison of level/intensity of coping strategies, PSS, AIS and HADS across respondents with different suicidal attitudes by sex strata.

Variable	Suicidal Ideation			Suicide Attempts		
	No	Yes	*p* *	No	Yes	*p* *
Females, n	219	403		472	150	
Dispositional Coping						
ACS	2.33 (2.00–3.00)	2.33 (1.67–3.00)	0.017	2.33 (1.67–3.00)	2.33 (1.67–3.00)	0.536
EF	2.00 (1.33–2.83)	3.00 (2.33–3.67)	<0.001	2.67 (1.67–3.33)	3.33 (2.33–3.67)	<0.001
SSS	2.30 (1.70–3.00)	2.00 (1.00–2.70)	<0.001	2.30 (1.30–3.00)	2.00 (1.30–2.70)	0.090
Situational Coping						
ACS	2.67 (2.33–3.33)	2.33 (2.00–3.00)	<0.001	2.67 (2.00–3.00)	2.67 (2.00–3.00)	0.561
EF	2.67 (2.00–3.33)	3.33 (2.67–3.67)	<0.001	3.00 (2.33–3.67)	3.33 (2.67–4.00)	<0.001
SSS	2.33 (1.67–3.00)	2.33 (1.33–3.00)	0.007	2.33 (1.33–3.00)	2.33 (1.33–3.00)	0.484
PSS—SUM	19.00 (16.00–25.00)	27.00 (23.00–31.00)	<0.001	24.00 (18.00–28.00)	29.00 (24.00–32.00)	<0.001
PSS—STEN	6.00 (5.00–8.00)	9.00 (8.00–10.00)	<0.001	8.00 (6.00–9.00)	9.00 (8.00–10.00)	<0.001
AIS—SUM	6.00 (4.00–9.00)	11.00 (7.00–15.00)	<0.001	8.00 (5.00–11.00)	13.50 (10.00–17.00)	<0.001
HADS-A anxiety	8.00 (5.00–11.00)	13.00 (10.00–16.00)	<0.001	10.00 (6.00–13.25)	15.00 (12.00–18.00)	<0.001
HADS-R aggression	3.00 (2.00–4.00)	4.00 (3.00–6.00)	<0.001	4.00 (2.00–5.00)	5.00 (3.25–6.00)	<0.001
HADS-D depression	4.00 (2.00–7.00)	9.00 (5.00–12.00)	<0.001	6.00 (3.00–9.00)	11.00 (7.25–13.00)	<0.001
Males, n	78	93		145	26	
Dispositional Coping						
ACS	2.33 (1.67–3.25)	2.33 (1.67–2.67)	0.303	2.33 (1.67–3.00)	2.16 (1.75–3.33)	0.686
EF	1.67 (0.67–2.33)	2.33 (1.33–3.00)	<0.001	1.67 (1.00–2.67)	2.67 (2.00–3.33)	<0.001
SSS	1.70 (0.70–2.30)	2.00 (1.00–2.70)	0.114	1.70 (0.70–2.70)	1.30 (0.47–2.00)	0.214
Situational Coping						
ACS	3.00 (2.33–3.33)	2.67 (2.00–3.33)	0.212	3.00 (2.33–3.33)	2.33 (2.00–3.00)	0.061
EF	2.00 (1.33–2.67)	2.67 (2.00–3.33)	<0.001	2.33 (1.67–3.00)	3.00 (2.67–3.33)	0.004
SSS	2.00 (1.00–2.67)	2.33 (1.67–3.33)	0.013	2.33 (1.00–3.00)	1.83 (0.67–2.58)	0.144
PSS—SUM	16.00 (12.00–19.75)	23.00 (18.00–28.00)	<0.001	18.00 (13.00–23.00)	26.00 (22.00–29.75)	<0.001
PSS—STEN	5.00 (4.00–6.75)	8.00 (6.00–9.00)	<0.001	6.00 (4.00–8.00)	8.00 (7.00–9.00)	<0.001
AIS—SUM	5.00 (3.00–7.00)	9.00 (5.00–12.00)	<0.001	5.00 (3.00–9.00)	12.00 (6.50–15.00)	<0.001
HADS-A anxiety	5.00 (2.00–9.00)	9.00 (7.00–13.00)	<0.001	7.00 (4.00–10.00)	11.50 (10.00–14.00)	<0.001
HADS-R aggression	2.00 (1.25–4.00)	4.00 (2.00–5.00)	0.002	3.00 (2.00–4.00)	4.00 (3.00–5.75)	0.006
HADS-D depression	3.00 (1.00–5.00)	7.00 (5.00–10.00)	<0.001	4.00 (2.00–7.00)	9.00 (7.00–12.75)	<0.001

Note: Data are presented as median and interquartile range q2 (q1–q3); * based on Mann–Whitney U test.

**Table 3:** Pairwise associations between level/intensity of coping strategies scores by sex strata—Spearman rank correlations.

Variable		Dispositional Coping			Situational Coping	
	ACS	EF	SSS	ACS	EF	SSS
	1	2	3	4	5	6
1		0.020	0.340 ***	0.590 ***	−0.110 **	0.220 ***
2	0.170 *		0.010	−0.150 ***	0.610 ***	0.010
3	0.290 ***	0.250 ***		0.240 ***	−0.060	0.700 ***
4	0.460 ***	−0.230 **	0.160 *		−0.080	0.250 ***
5	−0.010	0.690 ***	0.270 ***	−0.080		0.000
6	0.190 *	0.170 *	0.740 ***	0.200 **	0.300 ***	

Lower left triangle represents the results for males and upper right triangle for females. *** *p* < 0.001, ** *p* < 0.01, * *p* < 0.05.

**Table 4:** Association between negative emotions, place of living, PSS, HADS, AIS and EF dispositional strategy, and suicidality—logistic regression.

Variables	Suicidal Ideation		Suicide Attempts	
	Model 1	Model 2	Model 1	Model 2
	OR (95% CI)	OR (95% CI)	OR (95% CI)	OR (95% CI)
*HADS, PSS and AIS as categorical variables*				
Place of living				
Village	1 (ref.)	1 (ref.)	1 (ref.)	1 (ref.)
City	1.56 (1.11–2.21)	1.48 (1.04–2.11)	1.20 (0.82–1.77)	1.24 (0.84–1.84)
HADS				
No	1 (ref.)	1 (ref.)	1 (ref.)	1 (ref.)
Boundary state	1.79 (1.10–2.93)	1.82 (1.11–2.99)	2.47 (1.10–5.90)	2.33 (1.03–5.60)
Yes	3.37 (2.02–5.64)	3.59 (2.13–6.06)	6.21 (2.91–14.53)	6.29 (2.93–14.80)
PSS				
Low	1 (ref.)	1 (ref.)	1 (ref.)	1 (ref.)
Medium	1.21 (0.64–2.34)	1.31 (0.69–2.56)	0.88 (0.24–4.18)	0.85 (0.23–4.05)
High	2.08 (1.05–4.20)	2.26 (1.13–4.63)	0.84 (0.22–4.13)	0.86 (0.22–4.27)
AIS				
No	1 (ref.)	1 (ref.)	1 (ref.)	1 (ref.)
Yes	2.30 (1.57–3.37)	2.35 (1.60–3.46)	3.22 (2.04–5.21)	3.29 (2.07–5.35)
EF dispositional per 1	1.34 (1.12–1.61)	1.35 (1.12–1.62)	1.23 (1.00–1.53)	1.23 (0.99–1.53)
*HADS, PSS and AIS as quantitative variables*				
Place of living				
Village	1 (ref.)	1 (ref.)	1 (ref.)	1 (ref.)
City	1.59 (1.12–2.25)	1.49 (1.05–2.14)	1.27 (0.85–1.90)	1.33 (0.89–2.01)
HADS-A anxiety per 1	1.11 (1.04–1.17)	1.11 (1.04–1.18)	1.13 (1.05–1.21)	1.14 (1.06–1.22)
HADS-R aggression per 1	0.97 (0.87–1.09)	0.97 (0.86–1.08)	1.04 (0.91–1.19)	1.05 (0.91–1.20)
HADS-D depression per 1	1.09 (1.03–1.16)	1.09 (1.03–1.16)	1.06 (1.00–1.13)	1.07 (1.00–1.14)
PSS per 1	1.03 (0.99–1.08)	1.04 (0.99–1.09)	0.98 (0.93–1.03)	0.97 (0.92–1.02)
AIS per 1	1.08 (1.03–1.13)	1.08 (1.03–1.14)	1.14 (1.08–1.20)	1.14 (1.08–1.20)
EF dispositional per 1	1.25 (1.03–1.51)	1.25 (1.03–1.52)	1.16 (0.93–1.47)	1.18 (0.93–1.49)

Model 1: adjusted to place of living (village/city); emotional problems: HADS (No/Boundary state/Yes) or three domains of HADS given as continuous variables: Anxiety HADS-A, Aggression HADS-R and Depression HADS-D; PSS: low/medium/high or given as continuous variable (per 1 point), AIS (no/yes or given as continuous variable (per 1 point) and EF dispositional per 1 point; Model 2: adjusted to variables included in Model 1 and additionally to sex (Female/Male), Siblings (No/Yes), Age (categorical 16/17/18 years) and EF dispositional per 1 point; ref.—reference category.

## Data Availability

The data presented in this study are available on request from the corresponding author.

## References

[1] Osoby w Zamachach Samobójczych Według Grup Wieku (Suicides by Age Groups) In: GUS (Central Statistical Office-Poland) [Internet]. Warszawa. https://bdl.stat.gov.pl/BDL/metadane/cechy/3840?back=True#.

[2] Orri M., Galera C., Turecki G., Forte A., Renaud J., Boivin M., Tremblay R.E., Côté S.M., Geoffroy M.C. (2018). Association of childhood irritability and depressive/anxious mood profiles with adolescent suicidal ideation and attempts. JAMA Psychiatry.

[3] Hill R.M., Del Busto C.T., Buitron V., Pettit J.W. (2017). Depressive symptoms and perceived burdensomeness mediate the association between anxiety and suicidal ideation in adolescents. Arch. Suicide Res..

[4] CDC Centers for Disease Control and Prevention (CDC): WISQARS Leading Causes of Death Reports. CDC, Atlanta. https://www.cdc.gov.

[5] Kalin N.H. (2021). Anxiety, depression and suicide in youth. Am. J. Psychiatr..

[6] Kwon A., Song J., Yook K.H., Jon D.I., Jung M.H., Hong N., Hong H.J. (2020). Predictors of suicide attempts in clinically depressed Korean adolescents. Clin. Psychopharmacol. Neurosci..

[7] AlAzzam M., Abuhammad S., Tawalbeh L., Dalky H. (2021). Prevalence and correlates of depression, anxiety, and suicidality among high school students: A national study. J. Psychosoc. Nurs. Ment. Health Serv..

[8] Ang A.L., Wahab S., Abd Rahman F.N., Hazmi H., Md Yusoff R. (2018). Depressive symptoms in adolescents in Kuching, Malaysia: Prevalence and associated factors. Pediatr. Int..

[9] Madhavan S., Olino T., Klein D., Seeley J. (2021). Longitudinal predictors of suicidal ideation: Emerging to early adulthood. J. Psychiatr. Res..

[10] Geoffroy M., Orri M., Girard A., Perret L., Turecki G. (2021). Trajectories of suicide attempts from early adolescence to emerging adulthood: Prospective 11-year follow-up of a Candian cohort. Psychol. Med..

[11] Keliat B.A., Triana R., Sulistiowati N.M.D. (2019). The relationship between self-esteem, family relationships and social support as the protective factors and adolescent mental health. Humanit Soc. Sci. Rev..

[12] Detullio D., Kennedy T.D., Millen D.H. (2021). Adolescent aggression and suicidality: A meta-analysis. Aggress. Violent. Behav..

[13] Hartley C.M., Pettit J.W., Castellanos D. (2018). Reactive aggression and suicide-related behaviors in children and adolescents: A review and preliminary meta-analysis. Suicide Life Threat. Behav..

[14] Koyama E., Zai C.C., Bryushkova L., Kennedy J.L., Beitchman J.H. (2020). Predicting risk of suicidal ideation in youth using a multigene panel for impulsive aggression. Psychiatry Res..

[15] Miranda-Mendizabal A., Castellví P., Parés-Badell O., Alayo I., Almenara J., Alonso I., Blasco M.J., Cebria A., Gabilondo A., Gili M. (2019). Gender differences in suicidal behavior in adolescents and young adults: Systematic review and meta-analysis of longitudinal studies. Int. J. Public. Health.

[16] Benarous X., Consoli A., Cohen D., Renaud J., Lahaye H., Guilé J.M. (2019). Suicidal behaviors and irritability in children and adolescents: A systematic review of the nature and mechanisms of the association. Eur. Child. Adolesc. Psychiatry.

[17] Billings M.E., Hale L., Johnson D.A. (2019). Physical and social environment relationship with sleep health and disorders. Sleep. Med. Rev..

[18] Blank M., Zhang J., Lamers F., Taylor A.D., Hickie I.B., Merikangas K.R. (2015). Health correlates of insomnia symptoms and comorbid mental disorders in a nationally representative sample of US adolescents. Sleep.

[19] Werneck A.O., Vancampfort D., Oyeyemi A.L., Stubbs B., Silva D.R. (2018). Associations between TV viewing, sitting time, physical activity and insomnia among 100,839 Brazilian adolescents. Psychiatry Res..

[20] Fang H., Tu S., Sheng J., Shao A. (2019). Depression in sleep disturbance: A review on a bidirectional relationship, mechanisms and treatment. J. Cell Mol. Med..

[21] Narmandakh A., Roest A.M., de Jonge P., Oldehinkel A.J. (2020). The bidirectional association between sleep problems and anxiety symptoms in adolescents: A TRAILS report. Sleep. Med..

[22] Goldstone A., Javitz H.S., Claudatos S.A., Buysse D.J., Hasler B.P., de Zambotti M., Clark D.B., Franzen P.L., Prouty D.E., Colrain I.M. (2020). Sleep disturbance predicts depression symptoms in early adolescence: Initial findings from the adolescent brain cognitive development study. J. Adolesc. Health.

[23] Yang Y.T., Kaplan K.A., Zeitzer J.M. (2020). A comparison of sleep, depressive symptoms, and parental perceptions between U.S. and Taiwan adolescents with self-reported sleep problems. Sleep Adv..

[24] Haraden D.A., Mullin B.C., Hankin B.L. (2017). The relationship between depression and chronotype: A longitudinal assessment during childhood and adolescence. Depress. Anxiety.

[25] Walker W.H., Walton J.C., DeVries A.C., Randy J., Nelson R.J. (2020). Circadian rhythm disruption and mental health. Transl. Psychiatry.

[26] Baiden P., Tadeo S.K., Tonui B.C., Seastrunk J.D., Boateng G.O. (2020). Association between insufficient sleep and suicidal ideation among adolescents. Psychiatry Res..

[27] Zhang J., Paksarian D., Lamers F., Hickie I.B., He J., Merikangas K.R. (2017). Sleep patterns and mental health correlates in US adolescents. J. Pediatr..

[28] Maajida Aafreen M., Vishnu Priya V., Gayathri R. (2018). Effect of stress on academic performance of students in different streams. Drug. Invent. Today.

[29] Reiss F., Meyrose A.K., Otto C., Lampert T., Klasen F., Ravens-Sieberer U. (2019). Socioeconomic status, stressful life situations and mental health problems in children and adolescents: Results of the German BELLA cohort-study. PLoS ONE.

[30] Kim S., Sim S., Choi H. (2017). High stress, lack of sleep, low school performance, and suicide attempts are associated with high energy drink intake in adolescents. PLoS ONE.

[31] Cenkseven-Önder F. (2018). Social support and coping styles in predicting suicide probability among turkish adolescents. Univers. J. Educ. Res..

[32] Ford J.D., Charak R., Modrowski C.A., Kerig P.K. (2018). PTSD and dissociation symptoms as mediators of the relationship between polyvictimization and psychosocial and behavioral problems among justice-involved adolescents. J. Trauma. Dissociation.

[33] Yoon Y., Cederbaum J.A., Schwartz A. (2018). Childhood sexual abuse and current suicidal ideation among adolescents: Problem-focused and emotion-focused coping skills. J. Adolesc..

[34] Alix S., Cossette L., Hébert M., Cyr M., Frappier J.Y. (2017). Posttraumatic stress disorder and suicidal ideation among sexually abused adolescent girls: The mediating role of shame. J. Child. Sex. Abus..

[35] van den Heuvel M.W.H., Stikkelbroek Y.A.J., Bodden D.H.M., van Baar A.L. (2020). Coping with stressful life events: Cognitive emotion regulation profiles and depressive symptoms in adolescents. Dev. Psychopathol..

[36] Gould M.S., Lake A.M., Kleinman M., Galfalvy H., Chowdhury S., Madnick A. (2018). Exposure to suicide in high schools: Impact on serious suicidal ideation/behavior, depression, maladaptive coping strategies, and attitudes toward help-seeking. Int. J. Environ. Res. Public. Health.

[37] von Elm E., Altman D.G., Egger M., Pocock S.J., Gøtzsche P.C., Vandenbroucke J.P. (2008). STROBE Initiative. The Strengthening the Reporting of Observational Studies in Epidemiology (STROBE) statement: Guidelines for reporting observational studies. J. Clin. Epidemiol..

[38] Zigmond A.S., Snaith R.P. (1983). The hospital anxiety and depression scale. Acta Psychiatr. Scand..

[39] Majkowicz M., De Walden-Gałuszko K., Majkowicz M. (2000). Praktyczna Ocena Efektywności Opieki Paliatywnej-Wybrane Techniki Badawcze. Ocena Jakości Opieki Paliatywnej w Teorii i Praktyce.

[40] Mihalca A., Pilecka W. (2015). The factorial structure and validity of the Hospital Anxiety and Depression Scale (HADS) in Polish adolescents. Psychiatr. Pol..

[41] Cohen S., Kamarck T., Mermelstein R. (1983). A global measure of perceived stress. J. Health. Soc. Behav..

[42] Juczyński Z., Ogińska-Bulik N. (2009). Narzędzia Pomiaru Stresu i Radzenia Sobie ze Stresem.

[43] Lazarus R.S., Folkman S. (1984). Stress, Appraisal and Coping.

[44] Fornal-Pawłowska M., Wołyńczyk-Gmaj D., Szelenberger W. (2011). Validation of the Polish version of the Athens Insomnia Scale. Psychiatr. Pol..

[45] Yen C.F., King B.H., Chang Y.P. (2010). Factor structure of the Athens Insomnia Scale and its associations with demographic characteristics and depression in adolescents. J. Sleep. Res..

[46] Cha C.B., Franz P.J., Guzmán E.M., Glenn C.R., Kleiman E.M., Nock M.K. (2018). Annual research review: Suicide among youth-epidemiology, (potential) ethology, and treatment. J. Child. Psychol. Psychiatry.

[47] Harmer B., Lee S., Duong T.V.H., Saadabadi A. (2022). Suicidal Ideation.

[48] Ho T.C., Gifuni A.J., Gotlib I.H. (2022). Psychobiological risk factors for suicidal thoughts and behaviors in adolescence: A consideration of the role of puberty. Mol. Psychiatry.

[49] Pisinger V.S.C., Hawton K., Tolstrup J.S. (2017). Self-injury and suicide behavior among young people with perceived parental alcohol problems in Denmark: A school-based survey. Eur. Child. Adolesc. Psychiatry.

[50] Zygo M., Pawłowska B., Potembska E., Dreher P., Kapka-Skrzypczak L. (2019). Prevalence and selected risk factors of suicidal ideation, suicidal tendencies and suicide attempts in young people aged 13–19 years. Ann. Agric. Environ. Med..

[51] Brunner R., Kaess M., Parzer P., Fischer G., Carli V., Hoven C.W., Wasserman C., Sarchiapone M., Resch F., Apter A. (2014). Life-time prevalence and psychosocial correlates of adolescent direct self-injurious behavior: A comparative study of findings in 11 European countries. J. Child. Psychol. Psychiatry.

[52] Balázs J., Miklósi M., Keresztény Á., Hoven C.W., Carli V., Wasserman C., Apter A., Bobes J., Brunner R., Cosman D. (2013). Adolescent subthreshold-depression and anxiety: Psychopathology, functional impairment and increased suicide risk. J. Child. Psychol. Psychiatry.

[53] Avenevoli S., Swendsen J., He J.P., Burstein M., Merikangas K.R. (2015). Major depression in the national comorbidity survey-adolescent supplement: Prevalence, correlates, and treatment. J. Am. Acad. Child. Adolesc. Psychiatry.

[54] Breslau J., Gilman S., Stein B., Rude T., Gmelin T., Miller E. (2017). Sex differences in recent first-onset depression in an epidemiological sample of adolescents. Transl. Psychiatry.

[55] Miller L., Campo J.V. (2021). Depression in adolescents. N. Engl. J. Med..

[56] McDowell C.P., MacDonncha C., Herring M.P. (2017). Brief report: Associations of physical activity with anxiety and depression symptoms and status among adolescents. J. Adolescence.

[57] Elmasry N.M., Fouad A.A., Khalil D.M., Sherra K.S. (2016). Physical and verbal aggression among adolescent school students in Sharkia, Egypt: Prevalence and risk factors. Egyptian J. Psychiatry.

[58] Karriker-Jaffe K.J., Foshee V.A., Ennett S.T., Suchindran C. (2008). The development of aggression during adolescence: Sex differences in trajectories of physical and social aggression among youth in rural areas. J. Abnorm. Child. Psychol..

[59] Eliot L. (2021). Brain development and physical aggression: How a small gender difference grows into a violence problem. Curr. Anthropol..

[60] Stewart J.G., Shields G.S., Esposito E.C., Cosby E.A., Allen N.B., Slavich G.M., Auerbach R.P. (2019). Life stress and suicide in adolescents. J. Abnorm. Child. Psychol..

[61] Miller A.B., Eisenlohr-Moul T., Giletta M., Hastings P.D., Rudolph K.D., Nock M.K., Prinstein M.J. (2017). A within-person approach to risk for suicidal ideation and suicidal behavior: Examining the roles of depression, stress, and abuse exposure. J. Consult. Clin. Psychol..

[62] Anniko M.K., Boersma K., Tillfors M. (2019). Sources of stress and worry in the development of stress-related mental health problems: A longitudinal investigation from early- to mid-adolescence. Anxiety Stress Coping.

[63] Wilhsson M., Svedberg P., Högdin S., Nygren J.M. (2017). Strategies of adolescent girls and boys for coping with school-related stress. J. Sch. Nurs..

[64] Williams S.G., Borgogna N.S. (2020). Screening for stressful life events, cortisol, and perceived stress in a school setting. Ann. Pediatr..

[65] Maritta V., Ruthaychonnee S., Minna A. (2017). Survey of adolescents’ stress in school life in Thailand: Implications for school health. J. Child. Health Care..

[66] Salavera C., Usán P., Pérez S., Chato A., Vera R. (2017). Differences in happiness and coping with stress in Secondary Education students. Procedia Soc. Behav. Sci..

[67] Tugade M.M., Fredrickson B.L., Feldman Barrett L. (2004). Psychological resilience and positive emotional granularity: Examining the benefits of positive emotions on coping and health. J. Pers..

[68] Kerkhof G.A. (2017). Epidemiology of sleep and sleep disorders in The Netherlands. Sleep Med..

[69] Hsieh Y.-P., Lu W.-H., Yen C.-F. (2019). Psychosocial determinants of insomnia in adolescents: Roles of mental health, behavioral health, and social environment. Front. Neurosci..

[70] Donskoy I., Loghmanee D. (2018). Insomnia in adolescence. Med. Sci..

[71] Kansagra S. (2020). Sleep disorders in adolescents. Pediatrics.

[72] Lopez-Castroman J., Jaussent I. (2020). Sleep disturbances and suicidal behavior. Curr. Top. Behav. Neurosci..

[73] Trosman I., Ivanenko A. (2021). Classification and epidemiology of sleep disorders in children and adolescents. Child. Adolesc. Psychiatr. Clin. N. Am..

[74] Harris L.M., Huang X., Linthicum K.P., Bryen C.P., Ribeiro J.D. (2020). Sleep disturbances as risk factors for suicidal thoughts and behaviours: A meta-analysis of longitudinal studies. Sci. Rep..

[75] Fernandes S.N., Zuckerman E., Miranda R., Baroni A. (2021). When night falls fast: Sleep and suicidal behavior among adolescents and young adults. Child. Adolesc. Psychiatr. Clin. N. Am..

[76] Asarnow L.D., Mirchandaney R. (2021). Sleep and mood disorders among youth. Child. Adolesc. Psychiatr. Clin. N. Am..

[77] Inkelis S.M., Ancoli-Israel S., Thomas J.D., Bhattacharjee R. (2021). Elevated risk of depression among adolescents presenting with sleep disorders. J. Clin. Sleep. Med..

[78] McGlinchey E.L., Courtney-Seidler E.A., German M., Miller A.L. (2017). The role of sleep disturbance in suicidal and nonsuicidal self-Injurious behavior among adolescents. Suicide. Life Threat. Behav..

[79] Asarnow J.R., Bai S., Babeva K.N., Adrian M., Berk M.S., Asarnow L.D., Senturk D., Linehan M.M., McCauley E. (2020). Sleep in youth with repeated self-harm and high suicidality: Does sleep predict self-harm risk?. Suicide. Life Threat. Behav..

[80] Liu J.W., Tu Y.K., Lai Y.F., Lee H.C., Tsai P.S., Chen T.J., Huang H.C., Chen Y.T., Chiu H.Y. (2019). Associations between sleep disturbances and suicidal ideation, plans, and attempts in adolescents: A systematic review and meta-analysis. Sleep.

[81] Liu B.P., Wang X.T., Liu Z.Z., Wang Z.Y., Liu X., Jia C.X. (2019). Stressful life events, insomnia and suicidality in a large sample of Chinese adolescents. J. Affect. Disord..

[82] Li S.X., Chan N.Y., Yu M.W.M., Lam S.P., Zhang J., Chan J.W.Y., Li A.M., Wing Y.K. (2018). Eveningness chronotype, insomnia symptoms, and emotional and behavioural problems in adolescents. Sleep. Med..

[83] Gauvin G., Labelle R., Daigle M., Breton J.J., Houle J. (2019). Coping, social support, and suicide attempts among homeless adolescents. Crisis.

[84] Huang H.W., Wang R.H. (2019). Roles of protective factors and risk factors in suicidal ideation among adolescents in Taiwan. Public. Health Nurs..

[85] Mars B., Heron J., Klonsky E.D., Moran P., O’Connor R.C., Tilling K., Wilkinson P., Gunnell D. (2019). Predictors of future suicide attempt among adolescents with suicidal thoughts or non-suicidal self-harm: A population-based birth cohort study. Lancet Psychiatry.

[86] Beattie L., Kyle S.D., Espie C.A., Biello S.M. (2015). Social interactions, emotion and sleep: A systematic review and research agenda. Sleep. Med. Rev..

[87] Morales-Vives F., Dueñas J.M. (2018). Predicting suicidal ideation in adolescent boys and girls: The role of psychological maturity, personality traits, depression and life satisfaction. Spanish J. Psychol..

